# Ethnic inequalities in hospital admissions in England: an observational study

**DOI:** 10.1186/s12889-021-10923-5

**Published:** 2021-05-05

**Authors:** Jakob Petersen, Jens Kandt, Paul A. Longley

**Affiliations:** 1 Consumer Data Research Centre (CDRC), Department of Geography, University College London (UCL), Gower Street, London, WC1E 6BT UK; 2 The Bartlett Centre for Advanced Spatial Analysis (CASA), Gower Street, UCL, London, WC1E 6BT UK

**Keywords:** Health disparities, Ethnicity, Departments, hospital, Electronic health records

## Abstract

**Background:**

Ethnic inequalities in health are well-known and partly explained by social determinants such as poorer living and working conditions, health behaviours, discrimination, social exclusion, and healthcare accessibility factors. Inequalities are known both for self-reported health and for diseases such as diabetes, cardiovascular diseases, respiratory diseases, and non-specific chest pains. Most studies however concern individual diseases or self-reported health and do not provide an overview that can detect gaps in existing knowledge. The aim of this study is thus to identify ethnic inequalities in inpatient hospital admission for all major disease categories in England.

**Methods:**

Observational study of the inpatient hospital admission database in England enhanced with ethnicity coding of participants’ surnames. The primary diagnosis was coded to Level 1 of the Global Burden of Disease groups. For each year, only the first admission for each condition for each participant was included. If a participant was readmitted within two days only the first admission was counted. Admission risk for all major disease groups for each ethnic group relative to the White British group were calculated using logistic regression adjusting for age and area deprivation.

**Results:**

40,928,105 admissions were identified between April 2009 and March 2014. Ethnic inequalities were found in cardiovascular diseases, respiratory diseases, chest pain, and diabetes in line with previous studies. Additional inequalities were found in nutritional deficiencies, endocrine disorders, and sense organ diseases.

**Conclusions:**

The results of this study were consistent with known inequalities, but also found previously unreported disparities in nutritional deficiencies, endocrine disorders, and sense organ diseases. Further studies would be required to map out the relevant care pathways for ethnic minorities and establish whether preventive measures can be strengthened.

**Supplementary Information:**

The online version contains supplementary material available at 10.1186/s12889-021-10923-5.

## Introduction

Reducing inequalities in health has explicitly been part of the government agenda in the United Kingdom (UK) since 1997 [1, 2]. Ethnic inequalities in health are mainly caused by poorer living and working conditions, discrimination, marginalisation and differences in health and health seeking behaviours [3–7]. In exceptional cases, some groups are genetically predisposed to certain diseases such as sickle cell anaemia [3, 7]. Major conditions with known ethnic inequalities include diabetes, respiratory diseases, cardiovascular diseases, and non-specific chest pains [3–7]. National Health Service (NHS) hospitals monitor their use by ethnic group in the national database, Hospital Episode Statistics (HES), so that inequalities can be detected and ameliorated through adjustments in care provision and preventive action [8]. In this study we analyse hospital admission records by ethnic group across all major disease categories in the Global Burden of Disease (GBD) classification in 2009–2013 [9]. The study is intended to identify health inequalities across a wide spectrum of health conditions among in-patient admissions across England and its regions. There is an extensive literature on ethnic inequalities in health in the UK [3], but usually these pertain to individual diseases or self-reported health and do not provide an overview that can detect gaps in existing knowledge.

## Methods

Hospital admission records were obtained from NHS England’s Hospital Episode Statistics (HES), April 2009–March 2014 (financial years 2009–2013). Because some ethnic groups are relatively small, data on hospital admission for five years were analysed assuming that the 2011 Census population would be representative of the at-risk populations that year as well as two years before and two years after. Diagnoses in HES are coded to the 10th version of International Classification of Diseases (ICD10) system [10]. Data on the primary diagnosis for each admission were for the purpose of this study coded with definitions for the GBD conditions (Level 1). This high-level disease classification was developed for international comparison of health and healthcare factors, e.g. Cardiovascular diseases (2G) or Respiratory diseases (2H) [9]. If a patient was re-admitted within two days, only the first admission was counted. If a patient had more than one admission for the same condition in a year, only the first admission was kept. Area deprivation quintiles were coded at local authority level [11].

There is a growing literature about the use of names to impute ethnicity in studies where this information is not routinely collected or not otherwise available through data linkage [6, 12–14]. Software developed at University College London [13, 15] has been used in over 60 scientific studies and social equity audits in applications as diverse as accident and emergency department utilisation [16], residential segregation [17], labour market discrimination [18], and the composition of company boards [19]. To address problems with missing self-reported ethnicity information in HES, the Ethnicity Estimator (EE) software was deployed in this study [12, 13]. To retain full anonymity, this step was carried out in an air-gapped, secure data facility by NHS Digital linking name information in the Patient Demographic Service to HES. The sensitivity of the EE software in predicting the self-reported ethnic group was calculated. This study was part of a bigger project that reconciled ethnicity data within NHS over time (1999–2014) and between NHS, Censuses 2001 and 2011, and aggregation of ethnic groups in census outputs for small areas. “Arab” was grouped with “Other” in census outputs as there is no category for “Arab” in the NHS ethnicity classification. “Black other” was grouped with “Other” to reconcile how coded and aggregated in Census data tables for small areas. Preliminary work showed that surname imputation tends to inflate the White Irish group relative to self-reported data and the admission results for White Irish with imputed data are not shown.

The odds ratios of admission for different ethnic minorities were estimated with White British group as reference using logistic regression controlling for age in 20-year bands between 0 and 99 years of age and area deprivation quintile for each condition and all-cause hospitalisation. The analyses were repeated without surname imputation for sensitivity.

To identify geographic inequalities, the incidence of all-cause hospitalisation was calculated at local authority level and standardised by age and sex using the Census 2011 denominators and 2013 European Standard Population weights [20]. Counts below 20 cases were suppressed. Ethical approval was obtained from Bromley REC (Reference: 13/LO/1355) for analyses of patient-level HES data. The HES data licence reference is DARS-NIC-28051-Q3K7L.

## Results

There was a total of 40,928,105 admissions of 24,997,325 patients in 2009/13 of which 9.7% had missing self-reported ethnicity (Table 1). The total number of admissions by condition can be found in Table 2.

**Table 1:** NHS-recorded and EE-imputed ethnicity on HES admissions, 2009–2013

Ethnic Group	NHS-recorded	%	NHS-recorded with EE imputed	%
Asian Other	420,374	1.0	466,531	1.1
Bangladeshi	224,393	0.6	251,056	0.6
Chinese	117,965	0.3	140,002	0.3
Indian	748,626	1.8	862,019	2.1
Pakistani	742,705	1.8	856,327	2.1
Black African	498,148	1.2	572,557	1.4
Black Caribbean	377,545	0.9	395,783	1.0
Other	907,138	2.2	939,075	2.3
White Other	1,623,917	4.0	1,903,032	4.7
White British	30,610,616	74.8	33,540,602	82.0
White Irish	273,845	0.7	466,241	1.1
Mixed	409,095	1.0	409,095	1.0
Missing	3,973,738	9.7	125,785	0.3
Total	40,928,105	100	40,928,105	100

**Table 2:** Total number of admissions by Global Burden of Disease (Level 1) category in England, 2009–2013

GBD Level 1 Conditions	Freq.	%
1A Infectious and parasitic diseases	828,652	2.0
1B Respiratory infections	1,458,212	3.6
1C Maternal conditions	4,469,195	10.9
1D Perinatal conditions	455,236	1.1
1E Nutritional deficiencies	330,211	0.8
2A Malignant neoplasms	1,566,246	3.8
2B Other neoplasms	981,990	2.4
2C Diabetes mellitus	160,722	0.4
2D Endocrine disorders	515,230	1.3
2E Neuro-psychiatric conditions	1,965,073	4.8
2F Sense organ diseases	2,078,647	5.1
2G Cardiovascular diseases	2,699,978	6.6
2H Respiratory diseases	1,485,766	3.6
2I Digestive diseases	4,994,943	12.2
2 J Genito-urinary diseases	3,166,208	7.7
2 K Skin diseases	1,124,447	2.7
2 L Musculoskeletal diseases	3,981,799	9.7
2 M Congenital anomalies	307,166	0.8
2 N Oral conditions	1,187,828	2.9
30 Injuries	3,140,995	7.7
X102 Nonspecific chest pain	882,626	2.2
X176 Contraceptive and procreative management	150,778	0.4
X251 Abdominal pain	945,531	2.3
X257 Other aftercare	551,236	1.3
X259 Residual codes – unclassified	402,126	1.0
XR Symptoms signs and abnormal clinical and laboratory findings	528,269	1.3
XZ Factors influencing health status and contact with health services	568,870	1.4
Other	126	0.0
Total	40,928,105	100

The regression analyses showed a diverse picture where ethnic minorities have significantly higher admission for some conditions and lower for others (Table 3). These include higher risks for e.g. cardiovascular diseases for Bangladeshi and Pakistani or higher diabetes admission risk for the Black Caribbean group. It also showed that Pakistani, Other, and White other had higher admission risk than the White British group for most conditions, while Chinese and mixed ethnicity had significantly lower admission risk. Nutritional deficiencies were particular high for Bangladeshi, Pakistani, and Other (the dominant nutritional deficiency for all ethnic groups was iron deficiency anaemia). Endocrine disorders were higher in Black African, Black Caribbean, and Other (the dominant endocrine disorder for these groups were sickle-cell disorders). Sense organ diseases were higher in Other and South Asian groups (the dominant sense organ condition for all ethnic groups was cataracts). Non-specific chest pain admission, an early symptom of cardiovascular disease, was notably also very high in South Asians and the Other group. The risks deattenuated in analyses adjusted for area deprivation quintile in addition to age, especially for high-risk groups, which indicates that higher admission risk was partly explained by residence in deprived regions (Table 4).

**Table 3:**
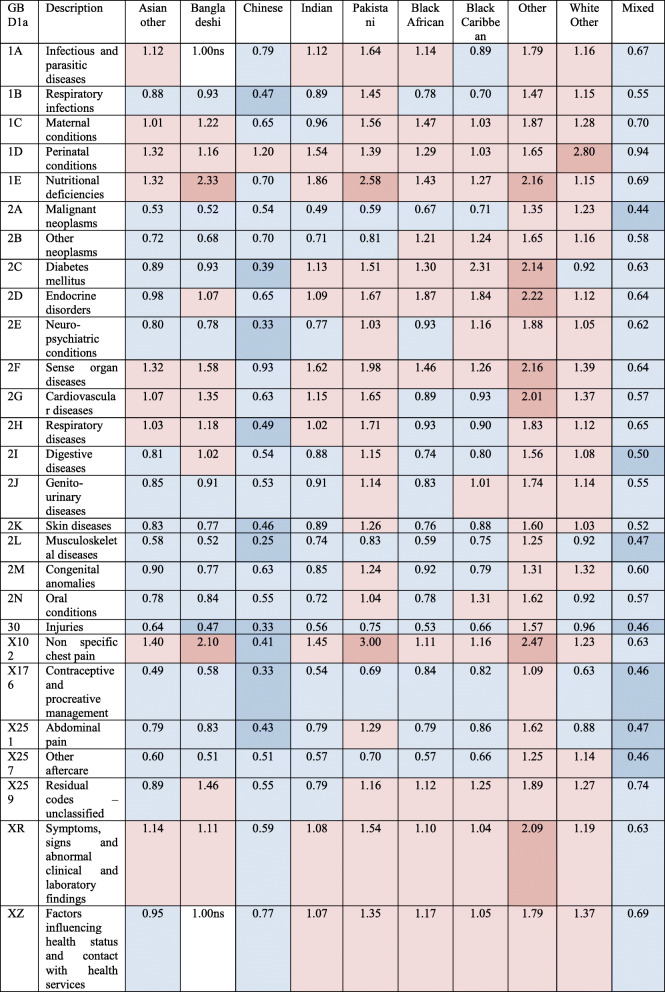
Age-adjusted odds ratios of admission incidence relative to White British group. Blue shading shows odds ratios significantly below White British group; red shading above. Darker tones show less than half and more than twice, respectively. Non-significant (ns) results shown on background. NHS-recorded ethnicity replaced with EE prediction where missing

**Table 4:**
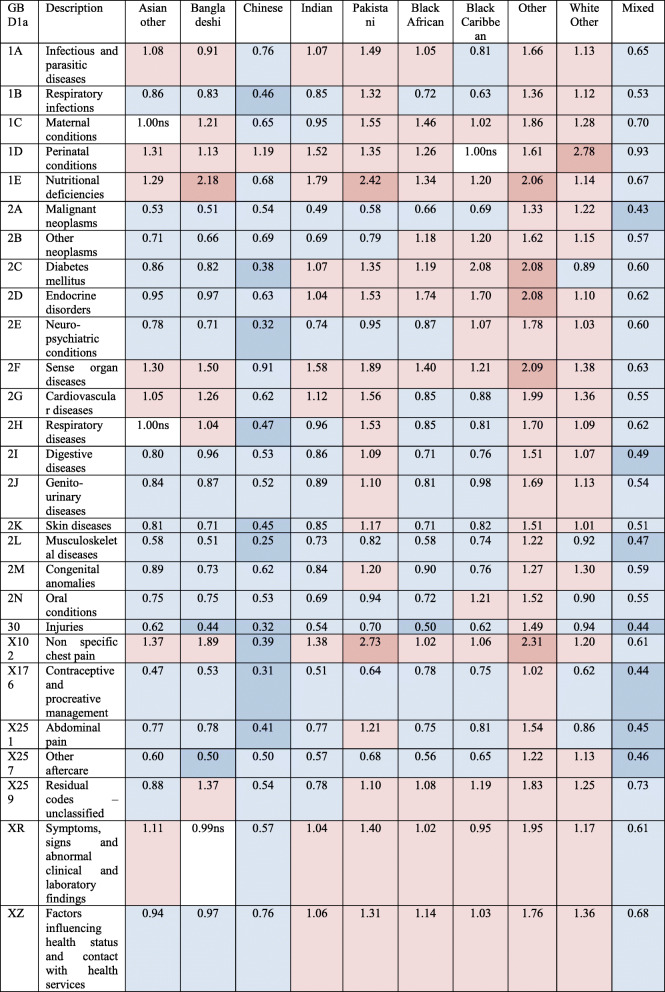
Age- and deprivation-adjusted odds ratios of admission incidence relative to White British group. Blue shading shows odds ratios significantly below White British group; red shading above. Darker tones show less than half and more than twice, respectively. Non-significant (ns) results shown on background. NHS-recorded ethnicity replaced with EE prediction where missing

**Fig. 1:**
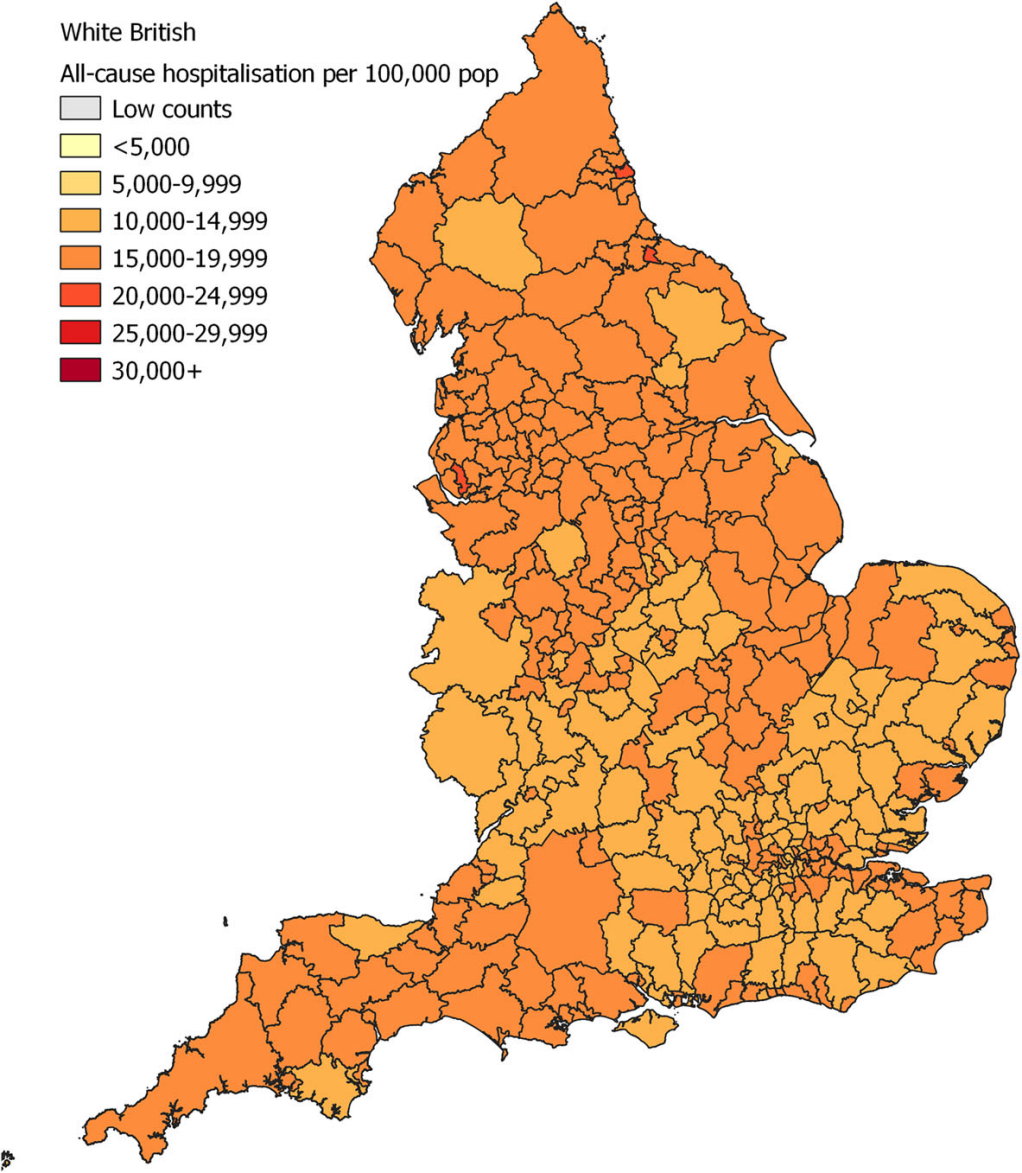
Map of all-cause hospitalisation per 100,000 population, 2009–2013, for White British

**Fig. 2:**
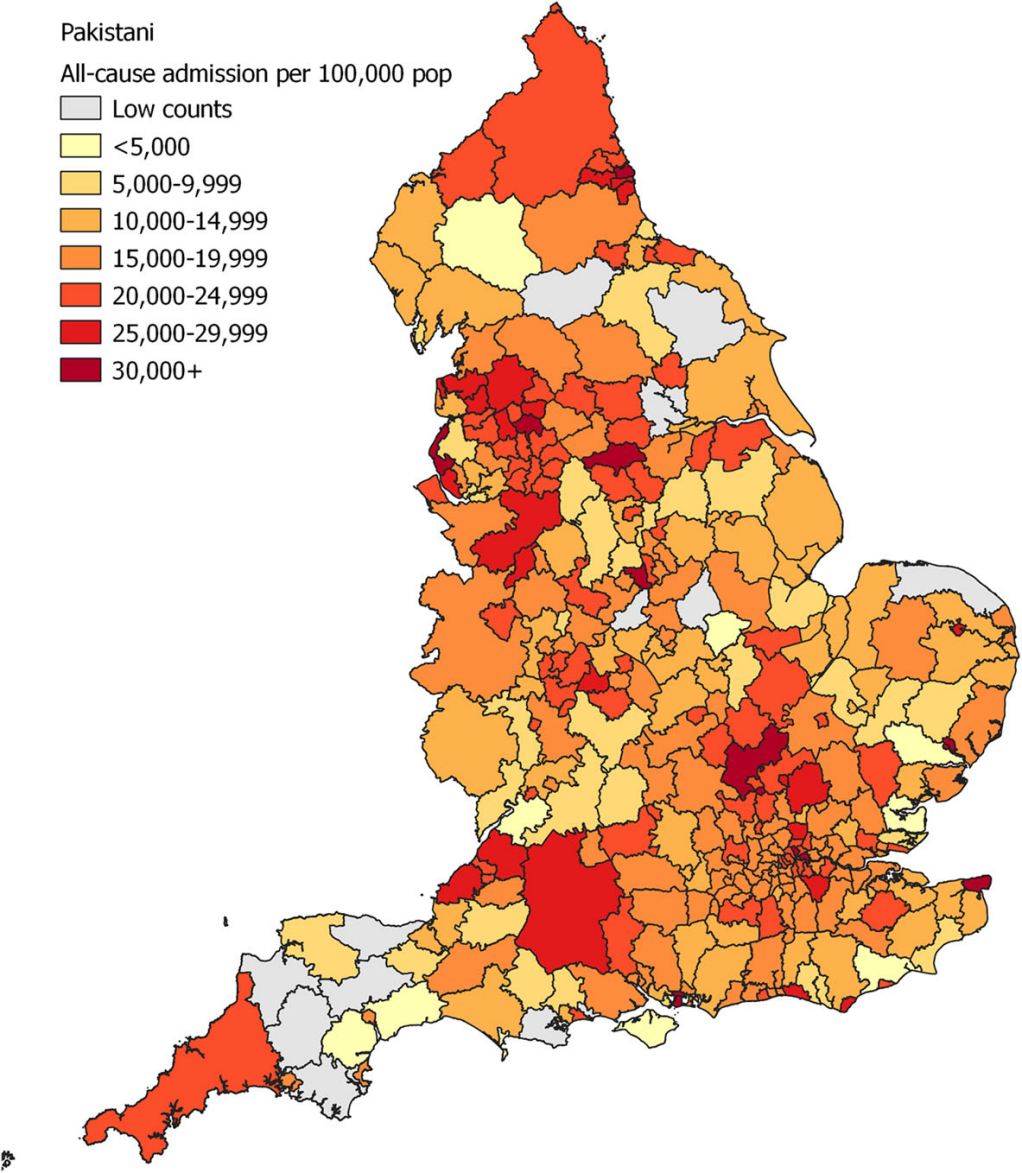
Map of all-cause hospitalisation per 100,000 population, 2009–2013, for Pakistani

**Fig. 3:**
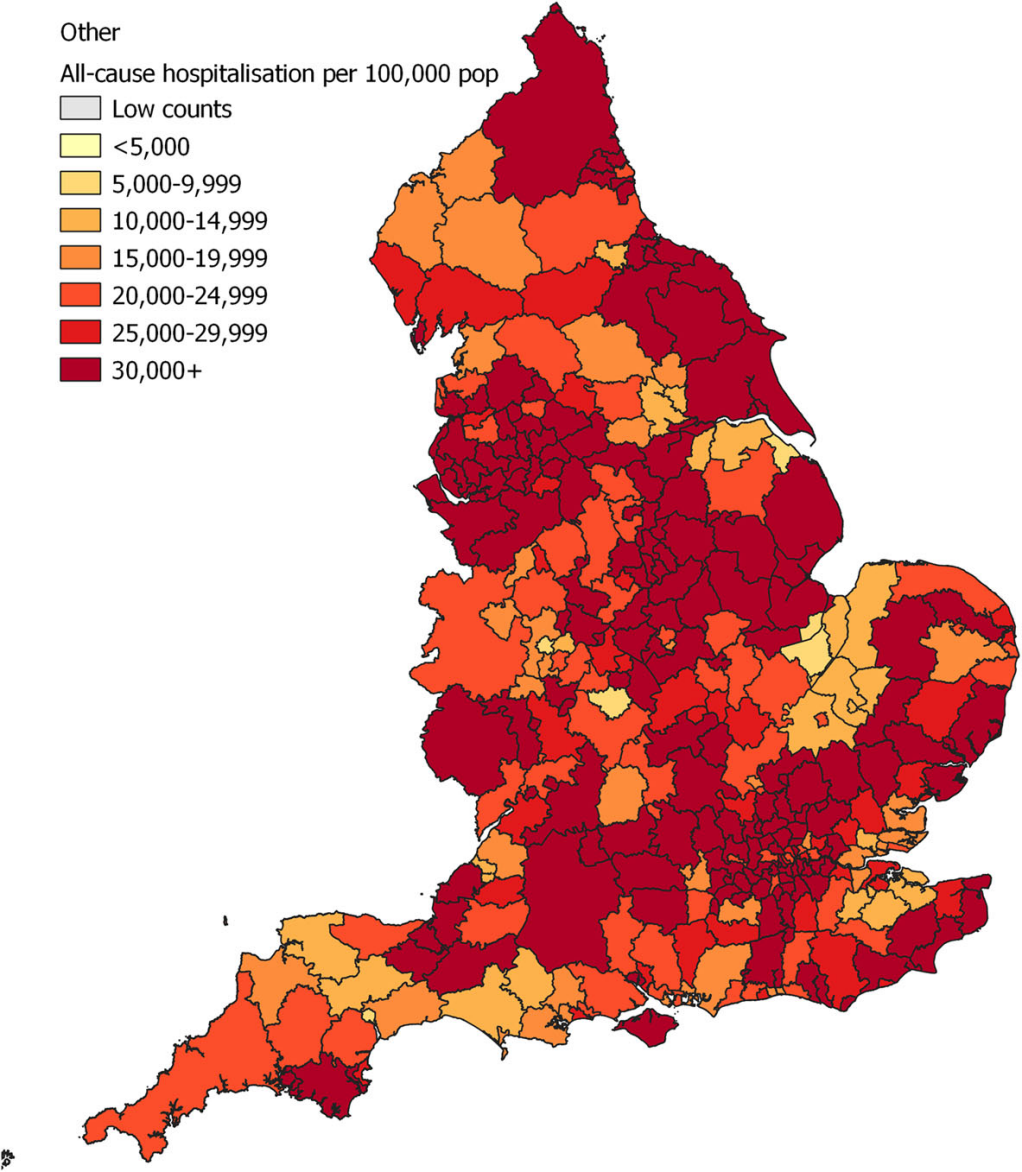
Map of all-cause hospitalisation per 100,000 population, 2009–2013, for Other ethnic group

For all-cause hospitalisation the risk was particularly high among patients with Pakistani (OR 1.28; 95% CI 1.28–1.29) and Other (1.83; 1.83–1.83) background compared to White British (Table 5; Table S3 for analysis without surname imputation). The maps showed that the risk of hospitalisation for White British was generally higher in Northern metropolitan areas (Fig. 1). For Pakistani (Fig. 2) and Other (Fig. 3), ethnic groups with higher risk of admission compared to White British, the regional distribution was more complex and widespread.

**Table 5:** Age- and deprivation- adjusted odds ratios (OR 95% CI) for all-cause hospitalisation. NHS-recorded ethnicity replaced with EE prediction where missing

Ethnic group	Case (N)	No case (N)	Age- adj. OR (95% CI)	P	Age-deprivation-adj. OR (95% CI)	P
White British	33,540,548	177,855,632	Ref	–	Ref	–
Asian Other	466,524	3,630,486	0.85 (0.85–0.86)	<.001	0.84 (0.84–0.84)	<.001
Bangladeshi	251,031	1,931,539	0.93 (0.92–0.93)	<.001	0.88 (0.87–0.88)	<.001
Chinese	140,011	1,757,504	0.51 (0.51–0.51)	<.001	0.50 (0.50–0.50)	<.001
Indian	862,039	6,116,471	0.88 (0.88–0.88)	<.001	0.86 (0.85–0.86)	<.001
Pakistani	856,301	4,705,109	1.28 (1.28–1.29)	<.001	1.21 (1.21–1.21)	<.001
Black African	572,582	4,316,123	0.94 (0.94–0.95)	<.001	0.90 (0.90–0.90)	<.001
Black Caribbean	395,784	2,559,296	0.90 (0.90–0.91)	<.001	0.86 (0.86–0.86)	<.001
Other	939,127	3,466,723	1.83 (1.83–1.83)	<.001	1.76 (1.76–1.76)	<.001
White Other	1,903,020	10,247,030	1.14 (1.14–1.14)	<.001	1.13 (1.12–1.13)	<.001
Mixed	409,131	5,555,264	0.55 (0.55–0.55)	<.001	0.54 (0.53–0.53)	<.001

When the records with missing ethnicity was replaced with EE categories, only 0.3% of admissions where without ethnicity coding. The sensitivity of the EE-prediction relative to NHS-recorded ethnicity varied by ethnic group: White British (89.6%), Pakistani (80.7%), Indian (73.9%), Chinese (71.0%), Bangladeshi (63.0%), Black African (54.9%), White Other (46.2%), White Irish (44.8%), Asian Other (20.7%), Black Caribbean (9.5%), Other (4.9%), and Mixed (0%). The regression analyses were repeated without the surname imputation for sensitivity and yielded similar results (Table S1, S2, S3, Supplementary materials).

## Discussion

Ethnic inequalities in health are well-known and partly explained by a combination of poorer living and working conditions and differences in health and health seeking behaviours [3–7]. The results of this study were consistent with known inequalities for diabetes, cardiovascular diseases, respiratory diseases, non-specific chest pains as well as previously unreported disparities in nutritional deficiencies, endocrine disorders, and sense organ diseases.

South Asians, Pakistani and Bangladeshi groups more than Indian, have relatively high risk of diabetes, coronary heart disease [21–24], asthma [5, 25], and certain gastrointestinal diseases [26]. The Pakistani group is also associated with higher risk of respiratory disease admission [27].

Black ethnic groups have higher risk of hypertension and diabetes [21]. Older studies showed higher risk of asthma admission among Black ethnic groups [28], while a more recent study did not find a higher risk of first asthma admission in Black ethnic groups compared to the White British group [25].

The results of this study confirmed previously reported risks for cardiovascular diseases, chest pain, and respiratory diseases with a few exceptions, e.g. Bangladeshis were not strongly associated with diabetes and respiratory disease admission and South Asians were not associated with gastrointestinal diseases. The reason for the latter is likely to be that the previous study only found an association for two less common gastrointestinal diseases, ulcerative colitis and Crohn’s disease [26], and that any association could have been swamped out by more common gastrointestinal disorders less specific to any particular ethnic group.

For diabetes, the Black Caribbean group had more than twice the admission risk of the White British group. The results also showed inequalities in areas that – to our knowledge - have not previously been reported in the UK, i.e. higher risk of nutritional deficiencies (Bangladeshi and Pakistani), endocrine disorders (Black African, Black Caribbean, and Other), and sense organ diseases (South Asian and Other).

Nutritional deficiencies can generally be classed as preventable and further studies would be required to identify relevant preventive measures for the identified ethnic groups, especially Bangladeshi, Pakistani and Other. The dominant nutritional deficiency was iron deficiency anaemia, which can be associated with dietary deficiency, increased loss or requirement, or malabsorption due to underlying health conditions [29].

This high-level analysis revealed ethnic inequalities in endocrine disorders for the Black African group, Black Caribbean group, and the Other group. The dominant endocrine disorder for these groups were sickle-cell disorders, which are rare genetic disorders typically found in persons with ancestors from sub-Saharan Africa [30].

Sense organ diseases were higher in Other and South Asian groups. The dominant sense organ condition for all ethnic groups was cataracts. This pattern is consistent with a UK survey of South Asian patients that showed higher cataract prevalence than the general population of the UK and the US [31].

The overall results show that ethnic disparities are wide ranging and involving many different care pathways. For some of these pathways there are well established secondary preventive care in the primary and community care sector, e.g. the monitory of patients with diabetes or heart conditions in general practice. This raises the question – in general and for further research - whether preventive infrastructures are used optimally by ethnic groups with known higher risk profiles, i.e. whether more could be done to prevent hospitalisation. This particular study was designed to detect ethnic inequalities in admissions overall and further studies would be required to narrow in on particular unmet needs and any specific barriers that ethnic groups may face navigating the healthcare system.

After more than two decades of policies and initiatives to explicitly reduce ethnic inequalities in the UK [1, 2], a review published in 2020 disconcertingly found evidence of persistent inequalities across many domains [3]. The review also found ethnic minorities less satisfied with the health services than the White British group and that they reported longer waiting times before being seen by a cancer specialist.

Chinese and mixed ethnic groups typically had much lower admission incidence that the White British group. The Chinese minority are known to have lower hospitalisation risk, good self-reported health [22], and lower mortality risk [32]. While the Chinese minority may have fewer health needs, they might also be less well recorded by NHS services compared to other groups due to a preference for traditional Chinese medicine [33].

Self-reported ethnicity information was missing for 7.3% of patients and was reduced to 0.2% by imputing ethnicity from patient surname to give a more complete representation. The sensitivity for the surname-derived information was better in some groups, e.g. more than 90% for White British and just above 50% for Black African. These biases must be acknowledged although limited since only 7.3% of patients in HES had no ethnicity data. The regression analyses in this study however yielded similar results with and without the surname imputation. The differences in sensitivity of the EE software are consistent with other studies and reflect factors such as differences in naming practices, sense of belonging, and migration history [13]. Although the sensitivity of the software is not equal for all groups, the alternatives in most cases are to analyse the data as complete case or imputing as if missing at random. The EE software is currently under development to improve its sensitivity and range not least for more recent expansions of the Census ethnicity classification with groups such as Arab and Roma.

A survey of cancer patients found consistency between NHS-recorded and self-reported ethnicity for 95% of participants [34]. This is a strength in relation to the use of self-reported ethnicity denominators from the Census.

## Conclusion

The study found stark disparities between ethnic minorities in hospitalisation for a sweep of conditions. Ethnic inequalities in health are well known for conditions such as cardiovascular diseases, respiratory diseases, and diabetes, but this study also found inequalities in nutritional deficiencies, endocrine disorders, and sense organ diseases.

Further studies would be required to map out the relevant care pathways for ethnic minorities and establish whether preventive measures can be strengthened.

## Supplementary Information

**Additional file 1: Table S1.** Age-adjusted odds ratios of admission incidence relative to white majority group. Blue shading shows odds ratios significantly below White British group; red shading above. Darker tones show less than half and more than twice, respectively. Non-significant (ns) results shown on white background. Ethnicity as recorded by NHS. **Table S2.** Age- and deprivation-adjusted odds ratios of admission incidence relative to white majority group. Blue shading shows odds ratios significantly below White British group; red shading above. Darker tones show less than half and more than twice, respectively. Non-significant (ns) results shown on white background. Ethnicity as recorded by NHS. **Table S3.** Age- and deprivation- adjusted odds ratios (OR 95% CI) for all-cause hospitalisation. Ethnicity as recorded by NHS.

## Data Availability

All the data that support the findings of this study are available from NHS Digital subject to ethnical and scientific approval of a study protocol. Researchers wishing to get access to the study data can visit this website: https://digital.nhs.uk/data-and-information/data-tools-and-services/data-services/hospital-episode-statistics. The authors of this study had no special access privileges others would not have.

## Abbreviations

UK: United Kingdom
NHS: National Health Service
HES: Hospital Episode Statistics
GBD: Global Burden of Disease
ICD10: 10th version of International Classification of Diseases
EE: Ethnicity Estimator

## References

[1] Mackenbach JP (2011). Can we reduce health inequalities? An analysis of the English strategy (1997–2010). J Epidemiol Community Health.

[2] Marmot M, Allen J, Boyce T, Goldblatt P, Morrison J. Health equity in England: the Marmot review 10 years on. The Health Foundation. 2020; https://www.health.org.uk/publications/reports/the-marmot-review-10-years-on. Accessed 10 Jul 2020.

[3] Chouhan K, Nazroo J (2020). Health inequalities. Ethnicity, race and inequality in the UK - state of the nation.

[4] Evandrou M, Falkingham J, Feng Z, Vlachantoni A (2016). Ethnic inequalities in limiting health and self-reported health in later life revisited. J Epidemiol Community Health.

[5] Wang S, Mak H-W. Generational health improvement or decline? Exploring generational differences of British ethnic minorities in six physical health outcomes. Ethn Health. 2018;0:1–14, DOI: 10.1080/13557858.2018.1557117. 10.1080/13557858.2018.1469736 29699405

[6] Petersen J, Kandt J, Longley PA (2021). Names-based ethnicity enhancement of hospital admissions in England, 1999–2013. Int J Med Inf.

[7] Kaufman JS, Rushani D, Cooper RS. Nature versus nurture in the explanations for racial/ethnic health disparities: parsing disparities in the era of genome-wide association studies. Oxford: Oxford University Press; 2018. https://oxford.universitypressscholarship.com/view/10.1093/oso/9780190465285.001.0001/oso-9780190465285-chapter-7. Accessed 10 Mar 2021.

[8] Alderwick H, Dixon J (2019). The NHS long term plan. The BMJ.

[9] Steel N, Ford JA, Newton JN, Davis ACJ, Vos T, Naghavi M, Glenn S, Hughes A, Dalton AM, Stockton D, Humphreys C, Dallat M, Schmidt J, Flowers J, Fox S, Abubakar I, Aldridge RW, Baker A, Brayne C, Brugha T, Capewell S, Car J, Cooper C, Ezzati M, Fitzpatrick J, Greaves F, Hay R, Hay S, Kee F, Larson HJ, Lyons RA, Majeed A, McKee M, Rawaf S, Rutter H, Saxena S, Sheikh A, Smeeth L, Viner RM, Vollset SE, Williams HC, Wolfe C, Woolf A, Murray CJL (2018). Changes in health in the countries of the UK and 150 English local authority areas 1990–2016: a systematic analysis for the global burden of disease study 2016. Lancet.

[10] WHO. ICD-10: International Statistical Classification of Diseases and Related Health Problems 10th Revision. 2016 https://icd.who.int/browse10/2016/en. Accessed 17 Jan 2020.

[11] Department for Communities and Local Government, 2015. The English Indices of deprivation 2015. Statistical Release; 2015. p. 1-38.

[12] Kandt J, Van Dijk J, Longley PA (2020). Family name origins and inter-generational demographic change in Great Britain. Am Geogr Soc.

[13] Kandt J, Longley PA (2018). Ethnicity estimation using family naming practices. PLoS One.

[14] Mateos P, Longley PA, O’Sullivan D (2011). Ethnicity and population structure in personal naming networks. PLoS One.

[15] Lakha F, Gorman DR, Mateos P (2011). Name analysis to classify populations by ethnicity in public health: validation of Onomap in Scotland. Public Health.

[16] Petersen J, Longley P, Gibin M, Mateos P, Atkinson P (2011). Names-based classification of accident and emergency department users. Health Place.

[17] Easton S, Pryce G (2019). Not so welcome here? Modelling the impact of ethnic in-movers on the length of stay of home-owners in micro-neighbourhoods. Urban Stud.

[18] Booth AL, Leigh A, Varganova E (2012). Does ethnic discrimination vary across minority groups? Evidence from a field experiment*. Oxf Bull Econ Stat.

[19] Bernile G, Bhagwat V, Yonker S (2018). Board diversity, firm risk, and corporate policies. J Financ Econ.

[20] European Commission. Revision of the European Standard Population — Report of Eurostat's task force. Luxembourg: Publications Office of the European Union, Methodologies and Working papers; 2013. 10.2785/11470.

[21] George J, Mathur R, Shah AD, Pujades-Rodriguez M, Denaxas S, Smeeth L, Timmis A, Hemingway H (2017). Ethnicity and the first diagnosis of a wide range of cardiovascular diseases: associations in a linked electronic health record cohort of 1 million patients. PLoS One.

[22] Health and Social Care Information Centre. Health Survey for England - 2004: Health of ethnic minorities 2006. https://digital.nhs.uk/data-and-information/publications/statistical/health-survey-for-england/health-survey-for-england-2004-health-of-ethnic-minorities-headline-results. .

[23] Hippisley-Cox J, Coupland C, Vinogradova Y, Robson J, Minhas R, Sheikh A, Brindle P (2008). Predicting cardiovascular risk in England and Wales: prospective derivation and validation of QRISK2. BMJ..

[24] Kristiansen M, Razum O, Tezcan-Güntekin H, Krasnik A (2016). Aging and health among migrants in a European perspective. Public Health Rev.

[25] Sheikh A, Steiner MFC, Cezard G, Bansal N, Fischbacher C, Simpson CR, et al. Ethnic variations in asthma hospital admission, readmission and death: a retrospective, national cohort study of 4.62 million people in Scotland. BMC Med. 2016;14:3. 10.1186/s12916-015-0546-6 PMC4710027 26755184

[26] Bhopal RS, Cezard G, Bansal N, Ward HJT, Bhala N (2014). Ethnic variations in five lower gastrointestinal diseases: Scottish health and ethnicity linkage study. BMJ Open.

[27] Bhopal R, Steiner MFC, Cezard G, Bansal N, Fischbacher C, Simpson CR, Douglas A, Sheikh A, SHELS Researchers (2015). Risk of respiratory hospitalization and death, readmission and subsequent mortality: Scottish health and ethnicity linkage study. Eur J Pub Health.

[28] Netuveli G, Hurwitz B, Levy M, Fletcher M, Barnes G, Durham SR, Sheikh A (2005). Ethnic variations in UK asthma frequency, morbidity, and health-service use: a systematic review and meta-analysis. Lancet.

[29] Goddard AF, James MW, McIntyre AS, Scott BB (2011). Guidelines for the management of iron deficiency anaemia. Gut..

[30] Kato G, Piel FBJ, Vichinsky E (2018). Sickle cell disease. Nat Rev Dis Primer.

[31] Rauf A, Malik R, Bunce C, Wormald R (2013). The British Asian community eye study: outline of results on the prevalence of eye disease in British Asians with origins from the Indian subcontinent. Indian J Ophthalmol.

[32] Bhopal RS, Gruer L, Cezard G, Douglas A, Steiner MFC, Millard A, Buchanan D, Katikireddi SV, Sheikh A (2018). Mortality, ethnicity, and country of birth on a national scale, 2001-2013: a retrospective cohort (Scottish health and ethnicity linkage study). PLoS Med.

[33] Sproston KA, Pitson LB, Walker E (2001). The use of primary care services by the Chinese population living in England: examining inequalities. Ethn Health.

[34] Saunders CL, Abel GA, El Turabi A, Ahmed F, Lyratzopoulos G (2013). Accuracy of routinely recorded ethnic group information compared with self-reported ethnicity: evidence from the English Cancer patient experience survey. BMJ Open.

