# Supplementary material for: Ethnic inequalities in hospital admissions in England: an observational study
Source: BMC Public Health. 2021 May 5;21:862. doi: 10.1186/s12889-021-10923-5 (PMC8097885; doi:10.1186/s12889-021-10923-5)
Supplement: Supplementary file 1 — Additional file 1: Table S1. Age-adjusted odds ratios of admission incidence relative to white majority group. Blue shading shows odds ratios significantly below White British group; red shading above. Darker tones show less than half and more than twice, respectively. Non-significant (ns) results shown on white background. Ethnicity as recorded by NHS. Table S2. Age- and deprivation-adjusted odds ratios of admission incidence relative to white majority group. Blue shading shows odds ratios significantly below White British group; red shading above. Darker tones show less than half and more than twice, respectively. Non-significant (ns) results shown on white background. Ethnicity as recorded by NHS. Table S3. Age- and deprivation- adjusted odds ratios (OR 95% CI) for all-cause hospitalisation. Ethnicity as recorded by NHS. [file 12889_2021_10923_MOESM1_ESM.docx]

Supplementary materials

**Table S1 Age-adjusted odds ratios of admission incidence relative to white majority group. Blue shading shows odds ratios significantly above White British group; red shading above. Darker tones show less than half and more than twice, respectively. Non-significant (ns) results shown on white background. Ethnicity as recorded by NHS.**

| GBD1a | Description | Asian other | Bangladeshi | Chinese | Indian | Pakistani | Black African | Black Caribbean | Other | White Other | White Irish | Mixed |
| --- | --- | --- | --- | --- | --- | --- | --- | --- | --- | --- | --- | --- |
| 1A | Infectious and parasitic diseases | 1.10 | 0.99ns | 0.74 | 1.07 | 1.58 | 1.09 | 0.91 | 1.86 | 1.09 | 0.65 | 0.71 |
| 1B | Respiratory infections | 0.88 | 0.92 | 0.45 | 0.87 | 1.42 | 0.75 | 0.71 | 1.53 | 1.09 | 0.70 | 0.59 |
| 1C | Maternal conditions | 1.00ns | 1.22 | 0.61 | 0.93 | 1.51 | 1.44 | 1.07 | 1.97 | 1.21 | 0.64 | 0.76 |
| 1D | Perinatal conditions | 1.31 | 1.17 | 1.11 | 1.51 | 1.34 | 1.25 | 1.07 | 1.73 | 2.66 | 1.21 | 1.02 |
| 1E | Nutritional deficiencies | 1.32 | 2.26 | 0.65 | 1.78 | 2.49 | 1.33 | 1.33 | 2.26 | 1.08 | 0.65 | 0.75 |
| 2A | Malignant neoplasms | 0.53 | 0.51 | 0.52 | 0.47 | 0.56 | 0.66 | 0.74 | 1.43 | 1.17 | 0.65 | 0.48 |
| 2B | Other neoplasms | 0.70 | 0.67 | 0.64 | 0.66 | 0.75 | 1.17 | 1.33 | 1.81 | 1.06 | 0.64 | 0.67 |
| 2C | Diabetes mellitus | 0.89 | 0.88 | 0.36 | 1.08 | 1.46 | 1.24 | 2.41 | 2.24 | 0.87 | 0.55 | 0.67 |
| 2D | Endocrine disorders | 0.98 | 1.06 | 0.62 | 1.06 | 1.64 | 1.87 | 1.94 | 2.35 | 1.06 | 0.79 | 0.69 |
| 2E | Neuro-psychiatric conditions | 0.80 | 0.77 | 0.31 | 0.73 | 0.98 | 0.89 | 1.21 | 1.99 | 0.99ns | 0.72 | 0.68 |
| 2F | Sense organ diseases | 1.30 | 1.50 | 0.88 | 1.53 | 1.89 | 1.37 | 1.35 | 2.30 | 1.30 | 0.63 | 0.71 |
| 2G | Cardiovascular diseases | 1.07 | 1.32 | 0.58 | 1.11 | 1.58 | 0.84 | 0.98 | 2.19 | 1.30 | 0.64 | 0.62 |
| 2H | Respiratory diseases | 1.03 | 1.17 | 0.46 | 0.97 | 1.65 | 0.87 | 0.93 | 1.92 | 1.04 | 0.76 | 0.71 |
| 2I | Digestive diseases | 0.80 | 0.98 | 0.49 | 0.84 | 1.08 | 0.70 | 0.84 | 1.66 | 1.00ns | 0.67 | 0.55 |
| 2J | Genito-urinary diseases | 0.85 | 0.90 | 0.49 | 0.87 | 1.09 | 0.79 | 1.07 | 1.85 | 1.07 | 0.62 | 0.61 |
| 2K | Skin diseases | 0.83 | 0.75 | 0.42 | 0.85 | 1.19 | 0.71 | 0.93 | 1.72 | 0.97 | 0.62 | 0.58 |
| 2L | Musculoskeletal diseases | 0.57 | 0.50 | 0.23 | 0.70 | 0.78 | 0.55 | 0.79 | 1.32 | 0.87 | 0.59 | 0.52 |
| 2M | Congenital anomalies | 0.88 | 0.75 | 0.58 | 0.79 | 1.17 | 0.85 | 0.83 | 1.43 | 1.17 | 0.72 | 0.69 |
| 2N | Oral conditions | 0.77 | 0.84 | 0.50 | 0.66 | 0.97 | 0.72 | 1.41 | 1.78 | 0.83 | 0.64 | 0.66 |
| 30 | Injuries | 0.63 | 0.45 | 0.30 | 0.53 | 0.70 | 0.49 | 0.68 | 1.65 | 0.90 | 0.64 | 0.49 |
| X102 | Non specific chest pain | 1.41 | 2.04 | 0.38 | 1.42 | 2.91 | 1.05 | 1.21 | 2.58 | 1.15 | 0.67 | 0.68 |
| X176 | Contraceptive and procreative management | 0.48 | 0.59 | 0.28 | 0.52 | 0.68 | 0.84 | 0.87 | 1.19 | 0.59 | 0.43 | 0.51 |
| X251 | Abdominal pain | 0.77 | 0.80 | 0.39 | 0.76 | 1.21 | 0.74 | 0.89 | 1.69 | 0.82 | 0.59 | 0.50 |
| X257 | Other aftercare | 0.59 | 0.50 | 0.48 | 0.55 | 0.67 | 0.54 | 0.68 | 1.29 | 1.09 | 0.66 | 0.49 |
| X259 | Residual codes –unclassified | 0.90 | 1.50 | 0.52 | 0.75 | 1.10 | 1.08 | 1.33 | 2.01 | 1.19 | 0.74 | 0.82 |
| XR | Symptoms, signs and abnormal clinical and laboratory findings | 1.15 | 1.09 | 0.57 | 1.05 | 1.49 | 1.06 | 1.08 | 2.19 | 1.14 | 0.64 | 0.67 |
| XZ | Factors influencing health status and contact with health services | 0.94 | 0.99ns | 0.71 | 1.04 | 1.31 | 1.14 | 1.10 | 1.90 | 1.28 | 0.76 | 0.76 |

**Table S2 Age- and deprivation-adjusted odds ratios of admission incidence relative to white majority group. Blue shading shows odds ratios significantly above White British group; red shading above. Darker tones show less than half and more than twice, respectively. Non-significant (ns) results shown on white background. Ethnicity as recorded by NHS.**

| GBD1a | Description | Asian other | Bangladeshi | Chinese | Indian | Pakistani | Black African | Black Caribbean | Other | White Other | White Irish | Mixed |
| --- | --- | --- | --- | --- | --- | --- | --- | --- | --- | --- | --- | --- |
| 1A | Infectious and parasitic diseases | 1.06 | 0.89 | 0.71 | 1.02 | 1.43 | 1.00ns | 0.83 | 1.72 | 1.06 | 0.64 | 0.68 |
| 1B | Respiratory infections | 0.85 | 0.82 | 0.44 | 0.82 | 1.28 | 0.68 | 0.65 | 1.41 | 1.06 | 0.68 | 0.56 |
| 1C | Maternal conditions | 0.99 | 1.20 | 0.60 | 0.92 | 1.49 | 1.42 | 1.06 | 1.94 | 1.20 | 0.64 | 0.76 |
| 1D | Perinatal conditions | 1.30 | 1.13 | 1.10 | 1.48 | 1.31 | 1.22 | 1.04 | 1.69 | 2.64 | 1.20 | 1.00ns |
| 1E | Nutritional deficiencies | 1.29 | 2.09 | 0.64 | 1.71 | 2.31 | 1.25 | 1.24 | 2.14 | 1.07 | 0.63 | 0.73 |
| 2A | Malignant neoplasms | 0.53 | 0.49 | 0.52 | 0.46 | 0.54 | 0.64 | 0.72 | 1.40 | 1.17 | 0.64 | 0.48 |
| 2B | Other neoplasms | 0.69 | 0.64 | 0.63 | 0.64 | 0.72 | 1.13 | 1.28 | 1.75 | 1.05 | 0.64 | 0.66 |
| 2C | Diabetes mellitus | 0.86 | 0.78 | 0.34 | 1.02 | 1.30 | 1.13 | 2.16 | 2.05 | 0.84 | 0.53 | 0.64 |
| 2D | Endocrine disorders | 0.95 | 0.95 | 0.60 | 1.01ns | 1.49 | 1.72 | 1.77 | 2.19 | 1.04 | 0.77 | 0.67 |
| 2E | Neuro-psychiatric conditions | 0.78 | 0.69 | 0.30 | 0.70 | 0.90 | 0.83 | 1.11 | 1.86 | 0.97 | 0.70 | 0.65 |
| 2F | Sense organ diseases | 1.27 | 1.40 | 0.86 | 1.48 | 1.75 | 1.30 | 1.27 | 2.20 | 1.29 | 0.62 | 0.69 |
| 2G | Cardiovascular diseases | 1.05 | 1.23 | 0.57 | 1.07 | 1.48 | 0.79 | 0.91 | 2.09 | 1.28 | 0.62 | 0.60 |
| 2H | Respiratory diseases | 0.99ns | 1.02 | 0.44 | 0.92 | 1.46 | 0.79 | 0.83 | 1.76 | 1.01 | 0.74 | 0.67 |
| 2I | Digestive diseases | 0.78 | 0.92 | 0.48 | 0.81 | 1.00 | 0.66 | 0.79 | 1.58 | 0.99 | 0.66 | 0.53 |
| 2J | Genito-urinary diseases | 0.83 | 0.85 | 0.48 | 0.85 | 1.03 | 0.75 | 1.01 | 1.79 | 1.05 | 0.61 | 0.60 |
| 2K | Skin diseases | 0.80 | 0.68 | 0.41 | 0.81 | 1.09 | 0.66 | 0.85 | 1.61 | 0.95 | 0.61 | 0.56 |
| 2L | Musculoskeletal diseases | 0.57 | 0.48 | 0.23 | 0.69 | 0.75 | 0.53 | 0.76 | 1.28 | 0.86 | 0.59 | 0.51 |
| 2M | Congenital anomalies | 0.87 | 0.71 | 0.57 | 0.77 | 1.11 | 0.81 | 0.78 | 1.37 | 1.15 | 0.71 | 0.67 |
| 2N | Oral conditions | 0.74 | 0.74 | 0.48 | 0.62 | 0.87 | 0.66 | 1.27 | 1.64 | 0.80 | 0.63 | 0.63 |
| 30 | Injuries | 0.62 | 0.42 | 0.29 | 0.51 | 0.65 | 0.46 | 0.63 | 1.56 | 0.89 | 0.62 | 0.48 |
| X102 | Non specific chest pain | 1.36 | 1.82 | 0.36 | 1.34 | 2.62 | 0.96 | 1.09 | 2.39 | 1.12 | 0.65 | 0.65 |
| X176 | Contraceptive and procreative management | 0.46 | 0.53 | 0.27 | 0.49 | 0.62 | 0.76 | 0.79 | 1.10 | 0.57 | 0.42 | 0.48 |
| X251 | Abdominal pain | 0.76 | 0.74 | 0.38 | 0.73 | 1.13 | 0.70 | 0.83 | 1.60 | 0.80 | 0.58 | 0.49 |
| X257 | Other aftercare | 0.58 | 0.48 | 0.48 | 0.54 | 0.65 | 0.52 | 0.66 | 1.26 | 1.08 | 0.66 | 0.49 |
| X259 | Residual codes –unclassified | 0.89 | 1.39 | 0.51 | 0.73 | 1.03 | 1.02 | 1.26 | 1.93 | 1.17 | 0.73 | 0.80 |
| XR | Symptoms, signs and abnormal clinical and laboratory findings | 1.11 | 0.97 | 0.55 | 1.00ns | 1.34 | 0.97 | 0.97 | 2.03 | 1.11 | 0.62 | 0.64 |
| XZ | Factors influencing health status and contact with health services | 0.93 | 0.94 | 0.70 | 1.02 | 1.25 | 1.10 | 1.06 | 1.85 | 1.27 | 0.75 | 0.75 |

**Table S3 Age- and deprivation- adjusted odds ratios (OR 95% CI) for all-cause hospitalisation. Ethnicity as recorded by NHS.**

| Ethnic group | Case (N) | No case (N) | Age-adj. OR (95% CI) | p | Age-deprivation-adj. OR (95% CI) | P |
| --- | --- | --- | --- | --- | --- | --- |
| White British | 30,610,479 | 180,785,701 | Ref | - | Ref | - |
| Asian Other | 420,406 | 3,676,605 | 0.84 (0.84-0.84) | <.001 | 0.83 (0.83-0.83) | <.001 |
| Bangladeshi | 224,355 | 1,985,215 | 0.91 (0.91-0.92) | <.001 | 0.85 (0.84-0.85) | <.001 |
| Chinese | 117,960 | 1,779,555 | 0.47 (0.47-0.48) | <.001 | 0.46 (0.46-0.46) | <.001 |
| Indian | 748,633 | 6,229,877 | 0.84 (0.84-0.84) | <.001 | 0.81 (0.81-0.81) | <.001 |
| Pakistani | 742,693 | 4,818,717 | 1.21 (1.20-1.21) | <.001 | 1.13 (1.13-1.13) | <.001 |
| Black African | 498,146 | 4,390,559 | 0.90 (0.90-0.90) | <.001 | 0.85 (0.85-0.85) | <.001 |
| Black Caribbean | 377,549 | 2,577,531 | 0.96 (0.95-0.96) | <.001 | 0.90 (0.90-0.90) | <.001 |
| Other | 907,208 | 3,498,642 | 1.96 (1.95-1.96) | <.001 | 1.87 (1.86-1.87) | <.001 |
| White Other | 1,623,894 | 10,526,156 | 1.06 (1.06-1.06) | <.001 | 1.04 (1.04-1.04) | <.001 |
| White Irish | 273,840 | 2,311,165 | 0.58 (0.58-0.59) | <.001 | 0.57 (0.57-0.57) | <.001 |
| Mixed | 409,126 | 5,555,269 | 0.61 (0.61-0.61) | <.001 | 0.59 (0.59-0.60) | <.001 |
